# The impact of TSH levels on clinical outcomes 14 days after frozen-thawed embryo transfer

**DOI:** 10.1186/s12884-020-03383-z

**Published:** 2020-11-10

**Authors:** Yuchao Zhang, Wenbin Wu, Yanli Liu, Yichun Guan, Xingling Wang, Liting Jia

**Affiliations:** 1 Department of Reproductive Medicine, the Third Affiliated Hospital of Zhengzhou University, No.7 Kangfuqian Street, Erqi, Henan Zhengzhou, China; 2 Neonatal screening center, the Third Affiliated Hospital of Zhengzhou University, Zhengzhou, Henan China

**Keywords:** TSH, 14 days, frozen-thawed embryo transfer, clinical pregnancy, miscarriage

## Abstract

**Background:**

The aim of this study was to investigate the impact of TSH levels on clinical outcomes 14 days after frozen-thawed embryo transfer.

**Methods:**

Blood samples were collected on the first visit to our department and 14 days after embryo transfer. Women were divided into three groups based on D14 TSH levels, which were compared to basal TSH levels in groups with different clinical outcomes. TSH levels between pregnant and nonpregnant women were also compared.

**Results:**

The clinical pregnancy rate in women with lower TSH levels 14 days after transfer was slightly but significantly lower (56%, *P* = 0.05) compared to those with higher TSH levels. Furthermore, TSH levels were significantly elevated 14 days after transfer compared to basal TSH levels in pregnant women and in women who successfully became pregnant (*P* < 0.001, respectively).

**Conclusions:**

Elevated TSH levels 14 days after embryo transfer compared to basal TSH levels seem to play a protective role and predict favorable clinical outcomes under specific conditions.

## Plain English

Some studies have observed increased TSH levels 14 days (D14 TSH) after fresh ET; however, few studies have focused on the impact of D14 TSH after frozen-thawed embryo transfer (FET) on clinical outcomes. In this study, we selected infertile women with basal TSH levels within the normal range and investigated the association between altered TSH levels and clinical outcomes after FET.

Among the 325 women analyzed, 125 women exhibited D14 TSH levels less than 2.5 mIU/L, while 139 women had D14 TSH levels between 2.5 and 4.2 mIU/L, and 61 women had TSH levels greater than 4.2 mIU/L. Interestingly, the pregnancy rate in women with lower TSH levels was significantly reduced, but the miscarriage rates among the three groups did not differ. In addition, no significant alterations were reported in women who were prepared for the transfer by natural cycles and failed to be pregnant and in women who were prepared for the transfer by stimulated cycles and aborted. In women being prepared for FET by natural cycles, higher D14 TSH levels may slightly predict miscarriage if age, serum AMH levels, basal TSH levels, and number of transferred embryos were adjusted.

In conclusion, elevated TSH levels 14 days after embryo transfer compared to basal TSH levels seemed to play a protective role and predicted favorable clinical outcomes under specific conditions.

## Background

Assisted reproductive technology (ART) has been widely used to aid infertile women in achieving successful pregnancy. Most studies tend to agree that there is a positive correlation between controlled ovarian stimulation (COS), during which period multiple dominant follicles develop and mature to improve chances for conception, and elevated TSH levels, which are believed by some experts to have adverse effects on ART outcomes. Poppe et al. [1] showed that serum TSH levels significantly and immediately increased after COS in patients receiving help from ART, and TSH levels had normalized during the follow-up period. Similarly, Du et al. [2] and Gracia et al. [3] also reported that serum TSH levels significantly increased after the start of GnRH-a, reaching peak levels after the injection of human chorionic gonadotropin (hCG). Furthermore, patients were more susceptible to COS when basal TSH levels were greater than 2.5 mIU/L. Most importantly, this study observed a higher clinical pregnancy rate when basal TSH levels were less than 2.5 mIU/L. However, whether moderately elevated TSH levels compromise ART clinical outcomes remains controversial [4–7]. Experts suggest that TSH concentrations < 2.5 mIU/L should be maintained during the ART procedure in infertile patients, especially in those with overt hypothyroidism [8–11]. Based on a population of more than eighteen thousand pregnant women in China, Chen et al. [4] clarified that high TSH levels preconception were associated with a small but significantly increased risk for overall adverse events, even within the normal nonpregnant range, and concluded that TSH < 2.5 mIU/L was more suitable for the assessment of women planning for a pregnancy.

On the other hand, Li et al. [12] found that in pregnant Chinese women, the upper limit of serum TSH reference in the first trimester was much higher than 2.5 mIU/L. The reference range for nonpregnant infertile women, which was supported by reproductive endocrinologists to be TSH < 4.2 mIU/L [13], could be used for assessment of pregnant women during 4 to 6 gestational weeks. However, one thing that should be mentioned is that the pregnant women included in the abovementioned studies were mostly at 4 gestational weeks at least, which is obviously different from 14 days after ET (D14 TSH).

Though some studies have reported increased D14 TSH after fresh ET, few studies have focused on the impact of D14 TSH after frozen-thawed embryo transfer (FET) on clinical outcomes, the ideal D14 TSH after FET, whether this parameter matters for clinical outcomes. The purpose of this study was to explore the role of D14 TSH in predicting clinical pregnancy or miscarriage after FET.

## Methods

### Patients

Infertile women from the Department of Reproductive Medicine of the Third Affiliated Hospital of Zhengzhou University receiving FET from May 1, 2019 to June 30, 2019 were initially recruited. Those without data on basal serum TSH levels, D14 TSH levels, and clinical outcomes were excluded. Women with basal TSH levels higher than 4.2 mIU/L were also excluded. Included women were divided into three groups based on D14 TSH levels (low group: ≤ 2.5 mIU/L; middle group: 2.5–4.2 mIU/L; and high group: > 4.2 mIU/L). This study was performed in accordance with the Code of Ethics in the Declaration of Helsinki.

### FET protocol

Either cleavage embryos or blastocysts were transferred in this study. Vitrified frozen embryos were thawed according to the standard procedure. Specifically, embryos were quickly released and immersed in thawing solution for 1 minute at 37℃. Then, embryos were moved into dilution solution for 3 minutes, followed by two steps in washing solution for 3 min each at room temperature. Thawed embryos were incubated for 2 h before transfer.

Patients were prepared for FET using either a stimulated or natural protocol. The natural protocol was supplied to women with normal menstrual cycles and regular ovulation. Ultrasonography and ovulation test paper were used to monitor and determine the ovulation time. Approximately 2–3 days before ovulation, serum luteinizing hormone (LH), estradiol (E_2_), and progesterone (P) were measured, and urine LH was measured every 4–6 h until it reached its peak. Then, HCG was administered by intramuscular injection at a dose of 10,000 IU, and endometrial conversion was performed the next day. Stimulated protocols involving hormone replacement therapy and ovulation induction were supplied to women with irregular ovulation or anovulation or to women with slow follicular development or luteal insufficiency. These women began taking letrozole on the 3rd -5th day of their menstrual cycle. Monitoring of ovulation and luteal support were performed in a manner similar to the natural protocol.

### TSH measurement

Before venipuncture, patients were required to stay calm for at least 30 minutes. Then, blood samples were collected on the morning of the 2nd -4th day of menstruation; samples were centrifuged at 3000 rpm for 10 minutes after at least half an hour. Then, the serum on the upper layer was used for analysis. Measurements of TSH, FT3 and FT4, as parts of the baseline panel of endocrine hormone measurements for first visitors, were conducted by electrochemical luminescence (ECLIA) on a Cobas 8000 (Roche Diagnostics, Germany). Daily internal quality controls were performed for all measurements.

### Statistical analysis

Continuous variables are expressed as the mean ± standard deviation, and categorical variables are expressed as the number or percentage. Either Student’s t-test or Mann-Whitney U test was used to compare differences between two continuous variables. Chi-square test was used for comparison of categorical variables. Paired t-test was used to compare alterations between basal TSH levels and D14 TSH levels in different groups categorized by transfer protocol. The impact of TSH levels on clinical outcomes was assessed by binary logistic regression. SPSS 22.0 was used for data analysis.

## Results

### Characteristics of included patients

**Fig. 1:**
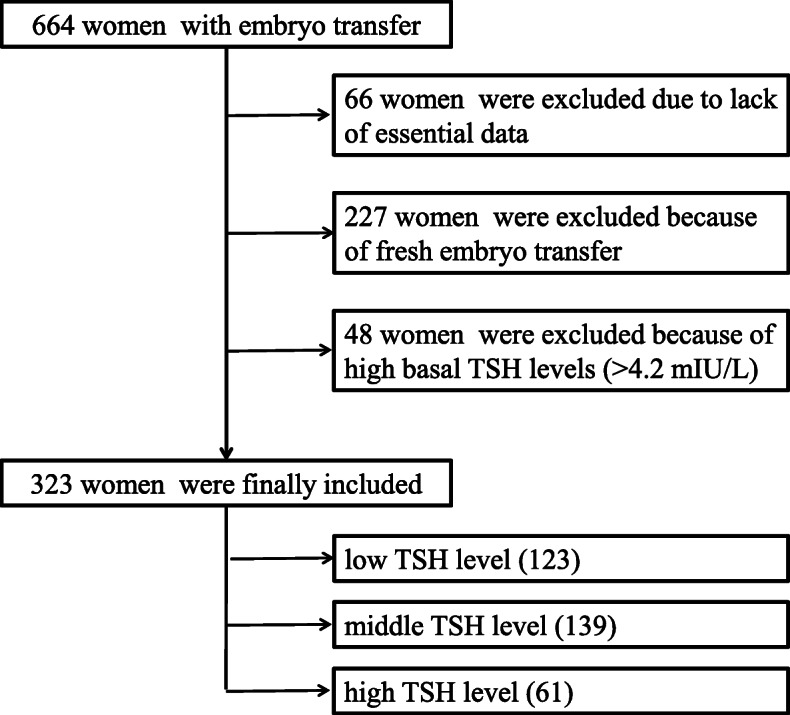
Flow chart of included subjects

A total of 664 women were initially included within the recruitment period. However, 66 women were excluded due to lack of essential data, and 227 women were excluded because the embryos were transferred at fresh cycles. Finally, 46 women were excluded because of elevated basal TSH levels (> 4.2 mIU/L). Among the 325 women remaining for analysis, 125 women had D14 TSH levels ≤ 2.5 mIU/L, 139 women had D14 TSH levels between 2.5 and 4.2 mIU/L, and 61 women had TSH levels > 4.2 mIU/L (Fig. 1). Average basal TSH levels were significantly increased compared to D14 TSH levels (*P* < 0.01). Age and BMI showed no significant differences in the different groups, while serum AMH levels in the middle level group were significantly higher compared to low- and high-level groups. The number of transferred blastocysts in different groups was not significantly different (*P* = 0.26). Interestingly, the pregnancy rate in women with lower TSH levels was statistically significantly lower (*P* = 0.05), but miscarriage rates among the three groups did not differ (*P* = 0.74) (Table 1).

**Table 1:** Characteristic of included patients based on D14 TSH levels

	Low TSH (*N* = 125)	Middle TSH (*N* = 139)	High TSH (*N* = 61)	*p*-value
Age (year)	30.73 ± 3.73	30.66 ± 3.64	30.11 ± 4.24	0.56
AMH (pmol/l)	23.74 ± 19.1	32.13 ± 26.8	28.23 ± 19.12	0.02
BMI (kg/m^2^)	23.35 ± 2.89	23.87 ± 3.19	23.63 ± 2.98	0.34
Basal TSH (IU/l)	2.01 ± 0.67	2.49 ± 0.86	2.75 ± 0.78	< 0.01
FT3 (pmol/l)	4.91 ± 0.52	4.70 ± 0.52	4.81 ± 0.68	0.01
FT4 (pmol/l)	16.95 ± 2.28	16.40 ± 2.41	16.29 ± 2.60	0.10
NO. of transferred blastocysts				
0	47(42.34%)	49(44.14%)	15(13.51%)	0.26
1	67(35.64%)	78(41.49%)	43(22.87%)	
2	11(42.31%)	12(46.15%)	3(11.54%)	
Clinical outcomes				
Pregnancy	70(56.00%)	98(70.50%)	38(62.30%)	0.05
Miscarriage	10(16.06%)	12(15.30%)	7(22.58%)	0.74

Data are expressed as the mean ± SD and n (%)

*D14 TSH* TSH levels 14 days after embryo transfer; *AMH* anti-mullerian hormone; *BMI* body mass index; *TSH* thyroid stimulating hormone

### Altered serum TSH levels between positive and negative clinical outcomes

Basal and D14 TSH levels in pregnant V.S. nonpregnant women and aborted V.S. nonaborted women were comparable. However, it should be noted that in both pregnant and non-aborted women, D14 TSH levels were significantly increased compared to basal TSH levels (*P* < 0.001). Interestingly, no significant alterations were reported in women who were prepared for the transfer by natural cycles and failed to become pregnant (*P* = 0.11) or in women who were prepared for the transfer by stimulated cycles and aborted (*P* = 0.45) (Table 2).

**Table 2:** Alterations in serum TSH levels between positive and negative clinical outcomes

		Frozen-warmed ET	Frozen-warmed ET with natural cycle	Frozen-warmed ET with stimulated cycles
D14 TSH	Pregnancy	3.12 ± 1.24	2.95 ± 1.12	3.21 ± 1.30
	Nonpregnancy	3.14 ± 3.03	3.55 ± 4.75	2.91 ± 1.28
	*p*	0.92	0.30	0.11
Basal TSH	Pregnancy	2.31 ± 0.81	2.20 ± 0.78	2.36 ± 0.83
	Nonpregnancy	2.44 ± 0.84	2.42 ± 0.77	2.45 ± 0.88
	*p*	0.15	0.15	0.46
Mean difference between paired D14 and basal TSH levels	Pregnancy	0.81 ± 1.25	0.74 ± 1.19	0.85 ± 1.28
	p	< 0.001	< 0.001	< 0.001
	Nonpregnancy	0.70 ± 2.90	1.13 ± 4.56	0.46 ± 1.20
	*p*	0.009	0.112	0.001
D14 TSH	Miscarriage	3.21 ± 1.31	3.37 ± 1.51	3.13 ± 1.23
	Ongoing pregnancy	3.10 ± 1.24	2.88 ± 1.04	3.22 ± 1.32
	*p*	0.66	0.20	0.78
Basal TSH	Miscarriage	2.35 ± 0.81	1.94 ± 0.65	2.55 ± 0.82
	Ongoing pregnancy	2.30 ± 0.82	2.24 ± 0.80	2.33 ± 0.83
	*p*	0.75	0.26	0.26
Mean difference between paired D14 and basal TSH levels	Miscarriage	0.86 ± 1.40	1.43 ± 1.63	0.58 ± 1.21
	*p*	0.002	0.022	0.45
	Ongoing pregnancy	0.80 ± 1.23	0.64 ± 1.08	0.90 ± 1.28
	*p*	< 0.001	< 0.001	< 0.001

Data are expressed as the mean ± SD

*D14 TSH* TSH levels 14 days after embryo transfer

### The impact of D14 TSH levels on clinical outcomes

Binary logistic regression was next employed to explore the impact of D14 TSH levels on clinical outcomes. Our results revealed that higher D14 TSH levels did not predict higher rates of pregnancy in women with FET, whether or not the confounders of age, serum AMH levels, basal TSH levels, and number of transferred embryos were adjusted. However, in women being prepared for FET by natural cycles, higher D14 TSH levels may slightly predict miscarriage if these confounders were adjusted (aOR = 0.53, *P* = 0.05) (Table 3).

**Table 3:** The impact of D14 TSH levels on clinical outcomes

	Pregnancy					Miscarriage			
	OR	*p*	adjusted OR	*p*		OR	*p*	adjusted OR	*p*
FET	0.99	0.62	0.99	0.84		0.93	0.66	0.91	0.58
FET with natural cycle	0.93	0.35	0.92	0.32		0.71	0.21	0.53	0.05
FET with stimulated cycle	1.21	0.11	1.20	0.15		0.76	0.78	1.16	0.51

*ET* embryo transfer; *OR* odds ratio

## Discussion

In this retrospective study, a total of 325 women were included for analysis. Herein, we described synchronously increased D14 TSH levels compared to basal TSH levels. In accordance with observations discovered by Du et al. [2] and Laura et al. [14], we found that in women with fresh ET, D14 TSH levels were significantly increased when basal TSH levels were less than 4.2 mIU/L (data not shown). Additionally, for the first time, we describe similar alterations in FET cycles, except in nonpregnant women who were prepared for FET using the natural protocol and in women who aborted and were prepared for FET using the stimulated protocol. Therefore, it remained to be determined whether very gentle stimulation of ovarian would lead to significantly increased TSH levels. For those with basal TSH levels higher than 4.2 mIU/L, levothyroxine was used to correct thyroid function, confounding interpretation of TSH levels in different ART phases when the data on TSH levels after treatment were missing.

As far as we know, this is the first study to investigate the association between clinical outcomes and D14 TSH levels after FET. Of note, even when D14 TSH levels were higher than 4.2 mIU/L, no detrimental effect was observed with regard to the clinical pregnancy rate. In contrast, a slightly but significantly lower clinical pregnancy rate was observed in women with D14 TSH levels ≤ 2.5 mIU/L. These results support the suggestion proposed by Konstantinos et al. [5] and Andrea et al. [15], claiming that moderately elevated TSH levels preconception (≤ 4.2 mIU/L) were not associated with adverse ART outcomes. Differences in either basal or D14 TSH levels between pregnancy and nonpregnancy, miscarriage and nonmiscarriage suggested that a single parameter, such as either basal or D14 TSH levels, may have no impact on clinical outcome.

A study performed by Shauna et al. [16] suggested that TSH rose significantly by the time pregnancy tests were taking, while it did not significantly change during the course of IVF treatment, which conflicted with previous studies [2, 3, 14]. In this study, D14 TSH levels were significantly increased compared to basal TSH levels in pregnant and non-aborted women, regardless of the transfer protocol. More interestingly, in women preparing for transfer using the natural protocol, no significant alterations were observed in those who did not achieve pregnancy, similar to the women who were prepared for transfer using the stimulated protocol who suffered from miscarriage. Taken together, these results may suggest a protective role for increased TSH levels in predicting clinical pregnancy after FET. A possible hypothesis to explain elevation of TSH in these women is the physiological response to pregnancy. Specifically, intact thyroid function is activated in response to elevated serum HCG, which produces more TSH and may contradict previous conclusions that in the early pregnancy (the first trimester) highly increased HCG leads to decreased TSH. In fact, the status of pregnancy resulted in hyperestrogenism, which in turn increased the secretion of serum thyroxine-binding globulin (TBG). The increased TBG then led to a temporary decrease in free thyroid hormones, which in turn resulted in increased TSH in a negative feedback manner. In other words, regardless of decreased median TSH levels in the first trimester [17], TSH elevation two weeks after embryo transfer may be a marker of successful activation of thyroid function in response to the initiation and maintenance of pregnancy.

A previous study claimed that patients with TSH levels > 2.5 mIU/L at the beginning of ovarian stimulation should be retested approximately 2 weeks after treatment and that this should be done at the same time as the pregnancy test to immediately identify patients requiring replacement therapy if pregnancy ensues. In fact, Zollner U et al. [18] also found that TSH concentrations, even those within normal range, were significantly higher in women with a clinical pregnancy than in nonpregnant women. However, whether elevated D14 TSH levels in FET cycles resulted from the terminal effects of ovarian stimulation, elevated effects of HCG, or physiological adjustment to pregnancy remained to be investigated. Increased TSH levels may be clinically irrelevant in consideration of clinical pregnancy rate, which was not reduced in women whose serum D14 TSH exceeded 4.2 mIU/L, and a high proportion of elevated D14 TSH levels was observed (18.77%), which was similar to the prevalence of SCH. Li et al. reported an upper limit of TSH levels higher than 2.5 mIU/L in the first trimester in Chinese pregnant women [12], so we based our observation on whether women with D14 TSH levels exceeding the threshold of 2.5 mIU/L should be treated or not in FET cycles.

Some limitations of this study should be taken seriously. First, the samples were collected from a short time period, and the small sample size may be a source of false positive results. Second, the number of transferred blastocysts, rather than the quality of embryos, was taken into consideration when adjusting for the impact of D14 TSH levels on clinical outcomes. Third, for women with basal TSH levels greater than 4.2 mIU/L, a more complex relationship among basal TSH, D14 TSH levels and clinical pregnancy may exist, so we excluded this population to simplify the results. Finally, the status of thyroid antibodies in these patients is unknown.

## Conclusions

In this study, we observed significantly elevated D14 TSH levels compared to basal TSH levels in pregnant and ongoing pregnant women, while no alterations were observed in D14 TSH levels in women who were prepared for FET using a natural cycle and did not achieve pregnancy or in those who were prepared for FET using a stimulated cycle and miscarried. These results may indicate a protective role for elevated D14 TSH levels in positive clinical outcome, but whether this elevation should be amended to maintain serum TSH levels below 2.5 mIU/L needs further investigation.

## Data Availability

The datasets used and/or analysed during the current study available from the corresponding author on reasonable request.

## Abbreviations

ART: Assistant reproductive technology
COS: Controlled ovarian stimulation
hCG: Human chorionic gonadotropin
D14 TSH: TSH levels 14 days after FET
FET: Frozen-thawed embryo transfer
LH: Luteinizing hormone
E_2_: Estradiol
P: Progesterone
ECLIA: Electrochemical luminescence
